# Identification and characterization of transcribed enhancers during cerebellar development through enhancer RNA analysis

**DOI:** 10.1186/s12864-023-09368-4

**Published:** 2023-06-26

**Authors:** Miguel Ramirez, Remi Robert, Joanna Yeung, Joshua Wu, Ayasha Abdalla-Wyse, FANTOM 5 Consortium, Daniel Goldowitz

**Affiliations:** 1 Centre for Molecular Medicine and Therapeutics, BC Children’s Hospital Research Institute, 950 W 28th Ave, V6H 3V5 Vancouver, BC Canada; 2 University of British Columbia, V6T 1Z4 Vancouver, BC Canada; 3 RIKEN, 2-1 Hirosawa, 351-0198 Wako, Saitama Japan

**Keywords:** Enhancers, Enhancer RNAs, Cerebellum development, Gene expression regulation, Epigenetics, Mouse

## Abstract

**Background:**

The development of the brain requires precise coordination of molecular processes across many cell-types. Underpinning these events are gene expression programs which require intricate regulation by non-coding regulatory sequences known as enhancers. In the context of the developing brain, transcribed enhancers (TEs) regulate temporally-specific expression of genes critical for cell identity and differentiation. Transcription of non-coding RNAs at active enhancer sequences, known as enhancer RNAs (eRNAs), is tightly associated with enhancer activity and has been correlated with target gene expression. TEs have been characterized in a multitude of developing tissues, however their regulatory role has yet to be described in the context of embryonic and early postnatal brain development. In this study, eRNA transcription was analyzed to identify TEs active during cerebellar development, as a proxy for the developing brain. Cap Analysis of Gene Expression followed by sequencing (CAGE-seq) was conducted at 12 stages throughout embryonic and early postnatal cerebellar development.

**Results:**

Temporal analysis of eRNA transcription identified clusters of TEs that peak in activity during either embryonic or postnatal times, highlighting their importance for temporally specific developmental events. Functional analysis of putative target genes identified molecular mechanisms under TE regulation revealing that TEs regulate genes involved in biological processes specific to neurons. We validate enhancer activity using in situ hybridization of eRNA expression from TEs predicted to regulate Nfib, a gene critical for cerebellar granule cell differentiation.

**Conclusions:**

The results of this analysis provide a valuable dataset for the identification of cerebellar enhancers and provide insight into the molecular mechanisms critical for brain development under TE regulation. This dataset is shared with the community through an online resource (https://goldowitzlab.shinyapps.io/trans-enh-app/).

**Supplementary Information:**

The online version contains supplementary material available at 10.1186/s12864-023-09368-4.

## Background

Brain development requires intricate regulation of gene expression programs whose co-ordination relies on non-coding regulatory sequences. Enhancers are a class of non-coding regulatory elements which serve as binding sites for transcription factors (TFs) and activate distal target gene transcription. In the context of brain development, enhancers are critical for regulating temporally- and cell type-specific gene expression [1]. Prenatal patterning and the development of numerous cell types is partially driven through the temporally and spatially restricted expression of TFs binding to enhancers. Transcriptional regulation by enhancers has also been shown to be critical for neuronal differentiation and maturation during the later stages of neuron development [2]. Despite the fundamental role of enhancers in brain development, our current knowledge is limited concerning the mechanisms by which they promote expression and the genes they regulate.

The discovery of transcription of non-coding RNAs at enhancer elements identified a subset of enhancers known as transcribed enhancers (TEs) [3]. The product of this transcription, termed enhancer RNAs (eRNAs), is highly correlated with markers of enhancer activity such as enhancer-associated histone marks (H3K27ac and H3K4me1), open chromatin conformation, TF binding, and the recruitment of transcriptional co-factors [4]. Large-scale reporter assays and perturbation studies have found that TEs are two- to three-fold more likely to show significant reporter activity than non-transcribed enhancer regions with associated histone marks [4–7]. More recent investigations of TEs indicate that eRNAs also contribute to the upregulation of gene expression in a context-dependent manner by establishing DNA accessibility through nucleosome displacement, stabilization of TF binding [8], recruitment and activation of transcriptional cofactors [9–13], release of transcriptional pausing [14], and promotion of cohesion-mediated enhancer-promoter contacts [15]. Additionally, TEs have been found to be enriched for disease-specific variants in a broad range of diseases including autoimmunity, cancer, infectious disease, and psychiatric and neurological disorders [16]. Overall, this evidence indicates that TEs are a subset of enhancers with a high likelihood to be functionally relevant.

In the context of development, TEs serve as binding sites for tissue-specific TFs resulting in the upregulation of gene expression. eRNA transcription at TEs is highly tissue specific and serves as markers of cell state [17]. TE elements are also enriched for cell-type and temporal-specific transcription factor binding sites of key regulators of cell differentiation and specification [4]. TEs and eRNAs have previously been found to regulate developmental transcriptional programs involved in skeletal muscle differentiation/myogenesis [18, 19], osteoclast development [20], T-cell and B-cell differentiation [15, 21], cardiac development [22] and embryonic stem cell differentiation [17]. In the context of the brain, the FANTOM5 project identified neural tissues and neurons as having a high abundance of cell-specific TE transcription [4]. Indeed, studies in neurons are prominent among those contributing to our understanding of enhancers and eRNA [3, 7, 23–25]. However, the role(s) of TEs has yet to be detailed in the context of the embryonic and early postnatal brain.

In our previous examination of enhancers in the cerebellum, we identified and characterized active enhancers during cerebellar development using post-translational histone modifications and identified enhancer signatures unique to embryonic and early postnatal stages [26]. These enhancers regulated genes with temporally and spatially restricted expression in the cerebellum which underpin molecular processes important for neuronal specification and differentiation. These findings are supported by previous examinations of enhancer activity in the postnatal cerebellum and through single-cell quantification of open chromatin conformation throughout mouse cerebellum development [2, 27]. Collectively, these studies demonstrate that the developing mouse cerebellum is an optimal setting to investigate gene expression regulatory mechanisms driving brain development. We predict that enhancers transcribed during the embryonic and early postnatal periods of brain development are temporally and spatially specific and regulate the expression of genes involved in neuronal development.

In this study, we identify TEs active during cerebellar development and characterize the developmental processes they regulate during embryonic and early postnatal stages. eRNA transcription is quantified using Cap Analysis of Gene Expression followed by sequencing (CAGE-seq) at 12 stages throughout embryonic and early postnatal cerebellar development. In combination with enhancer-associated histone modifications H3K4me1 and H3K27ac, we establish a compendium of robust cerebellar TEs. Temporal analysis of eRNA transcription identifies clusters of TEs that peak in activity during either embryonic or postnatal stages, highlighting their importance for temporally specific developmental events. A comparison with tissues from the FANTOM5 database indicates that robust cerebellar TE transcription is specific to the cerebellum. Putative gene targets are identified by correlating TE transcription with expression of cis-located genes. Functional analysis of target genes identify molecular mechanisms under TE regulation revealing that TEs regulate genes involved in biological processes specific to cells in the brain; while non-transcribed enhancers regulate genes involved in non-specific constitutive processes.

## Results

### Identification of cerebellar transcribed enhancers

**Fig. 1:**
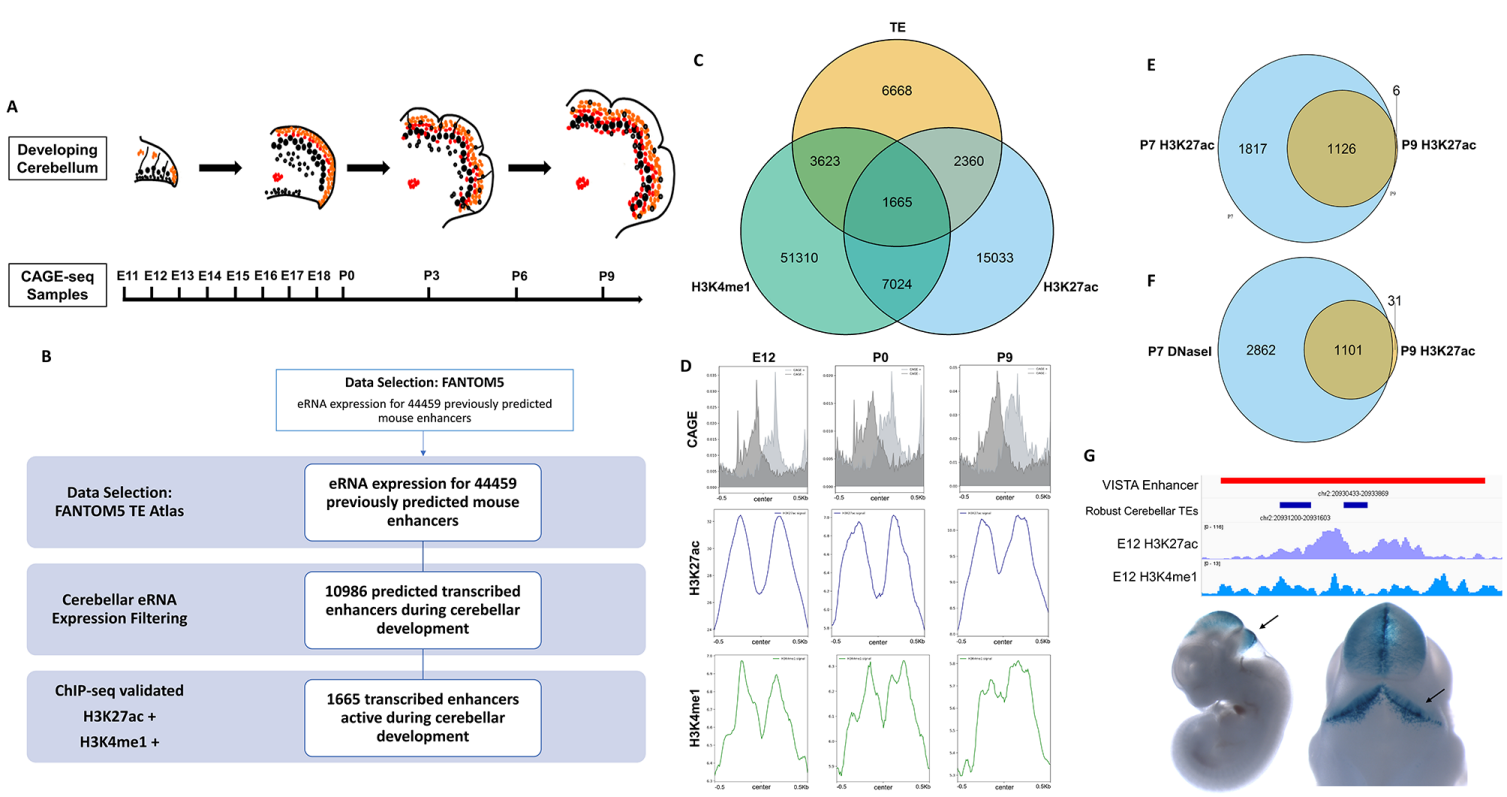
Identification of transcribed enhancers (TEs) in the developing cerebellum using eRNA transcription quantification and epigenomic profiling. **(A)** Timeline of cerebellar development and stages chosen for eRNA quantification through Cap Analysis of Gene Expression followed by sequencing (CAGE-seq). **(B)** Flow chart depicting the pipeline for identifying an atlas of robust cerebellar TEs. **(C)** Venn diagram displaying the number of TEs validated by H4K4me1 and H3K27ac ChIP-seq in the developing cerebellum profiled at E12, P0 and P9. **(D)** Profiles of mean normalized CAGE-seq count (top), H3K27ac ChIP-seq signal (middle) and H3K4me1 ChIP-seq signal (bottom) for robust cerebellar TEs relative to the centre of these elements. **(E)** Venn diagrams showing the overlap between cerebellar TEs with H3K27ac peak signal at P9 and at P7. **(F)** Venn diagram showing the overlap between cerebellar TEs with H3K27ac peak signal at P9 and DNase-seq peak signal at P7. **(G) Top**: Integrative Genomics Viewer (IGV) browser showing genomic locations for one enhancer with hindbrain activity from the VISTA Enhancer Database (mm1447) and two robust cerebellar TEs. Tracks for H3K27ac and H3K4me1 ChIP-seq signals at these coordinates are also displayed. **Bottom**: Images of LacZ enhancer reporter transgenic mouse for sequence mm1447 from the VISTA Enhancer Database. Rhombencephalon expression is driven by this enhancer sequence, as seen with LacZ staining (blue) of embryos. Images sourced from the VISTA Enhancer Database. TPM = transcripts per million

To identify transcribed enhancers (TEs) active during cerebellar development, eRNA expression was quantified from an atlas of TEs previously constructed by the FANTOM5 consortium based on bi-directional eRNA expression [4, 17]. This database consists of 44,259 TEs found to transcribe bi-directional eRNAs in mouse tissues and was quantified by Cap Analysis of Gene expression followed by sequencing (CAGE-seq). We focused on a CAGE-seq times series previously quantified in the developing cerebellum [28] (Fig. 1A). 10,986 active TEs were identified in the developmental time course from the analysis described in the Methods (i.e., eRNA transcription present at a minimum of 3 time points and at a level of > = 0.5 TPM). These are referred henceforth as cerebellar TEs (Fig. 1B). Robust cerebellar TEs are expressed at a higher level (a 2.37 fold increase on average) compared to non-robust cerebellar TEs at all stages examined during cerebellar development (Supplementary Fig. 1A).

Peak signals for enhancer associated histone marks H3K27ac and H3K4me1 have been found at TEs, serving as additional signals of open chromatin conformation and enhancer activity [4, 17]. To filter for a more robust set of cerebellar TEs with a higher likelihood of activity, cerebellar TE coordinates were overlapped with H3K27ac and H3K4me1 ChIP-seq data previously conducted at three time points throughout cerebellar development: E12, P0 and P9 [26]. We identified that 33% (3623/10,986) of cerebellar TEs overlapped with H3K4me1 peaks, 21.5% (2360/10,986) overlapped with H3K27ac peaks and 15.2% (1665/10,986) overlapped with both marks (Fig. 1C). The 1664 TEs overlapping with both H3K27ac and H3K4me1 peaks are considered robust cerebellar TEs (Supplementary Table 1).

eRNA expression and histone ChIP-seq signals were profiled at robust cerebellar TEs and cerebellar TEs as measures of validation. Robust cerebellar TEs showed a bimodal distribution of CAGE-tags flanking the centre of the sequences, indicating bi-directional transcription at these TEs (Fig. 1D). For cerebellar TEs, we observe a similar bimodal distribution, however the eRNA transcription is decreased at these sequences compared to robust cerebellar TEs (Supplementary Fig. 1B). H3K4me1 and H3K27ac profiles at robust cerebellar TEs also exhibited a bimodal distribution of ChIP-seq signal flanking the centre of the sequences, indicating deposition adjacent to these TEs (Fig. 1D). Previous studies have shown that eRNA as well as H3K4me1 and H3K27ac marks exhibit a similar bimodal distribution of signal at TEs when using P300 binding sites as the centre point [4]. Expectedly, we observed no histone signal at these time points for cerebellar TEs (Supplementary Fig. 1C). These results indicate that our analysis identifies TEs with transcriptional and epigenetic properties typically found at these elements.

We then conducted a confirmatory analysis to evaluate whether cerebellar TEs and robust cerebellar TEs can be identified by independent epigenomic datasets generated from the postnatal cerebellum. To do this, the same overlap analysis was performed with datasets from a previous study by Frank et al. (2015) [2] who used H3K27ac ChIP-seq and DNAse-seq in the postnatal cerebellum (P7) to investigate changes in chromatin conformation from postnatal to adult stages. The results of these overlaps are reported in Supplementary Fig. 1D. We identified **3968/10,986** cerebellar TEs overlapping with P7 H3K27ac peak coordinates and 3936/10,986 TEs overlapping with P7 DNase-seq peak coordinates. For robust cerebellar TEs, we identified 1528/1665 overlapping with P7 H3K27ac peaks and 1524/1665 overlapping with open chromatin regions at P7 defined by DNase-seq. These genomic locations of cerebellar TEs with H3K27ac signal or DNase-seq signal at P7 were then overlapped with the coordinates of the 1132/10,986 cerebellar TEs with H3K27ac signal in our samples at P9. We find that 99.5% (1126/1132) of the TEs with H3K27ac peak signal at P9 also had peak signal at P7, adding credence to our findings (Fig. 1E). We also find that 97% (1099/1132) of the TEs with H3K27ac peak signal at P9 also have DNase-seq peak signal at P7, indicating open chromatin conformation at these TEs (Fig. 1E). We then asked whether any of our robust cerebellar TEs overlapped enhancers with reporter activity in the embryonic brain cataloged in the VISTA Enhancer Database [29]. We identified 60 cerebellar TEs and 57 robust cerebellar TEs that overlapped with enhancers with activity in the rhombencephalon during embryonic development (Supplementary Table 2). Shown in Fig. 1G is an example of two robust cerebellar enhancers that overlap with an enhancer from the VISTA Enhancer Database (mm1447) with LacZ reporter signal in the developing cerebellum. Collectively, comparisons with previous literature and datasets indicate that robust cerebellar TEs represent viable candidate regulatory sequences likely to be active during cerebellar development.

### Cerebellar TE transcription exhibits temporally-dynamic and tissue-specific expression throughout development

**Fig. 2:**
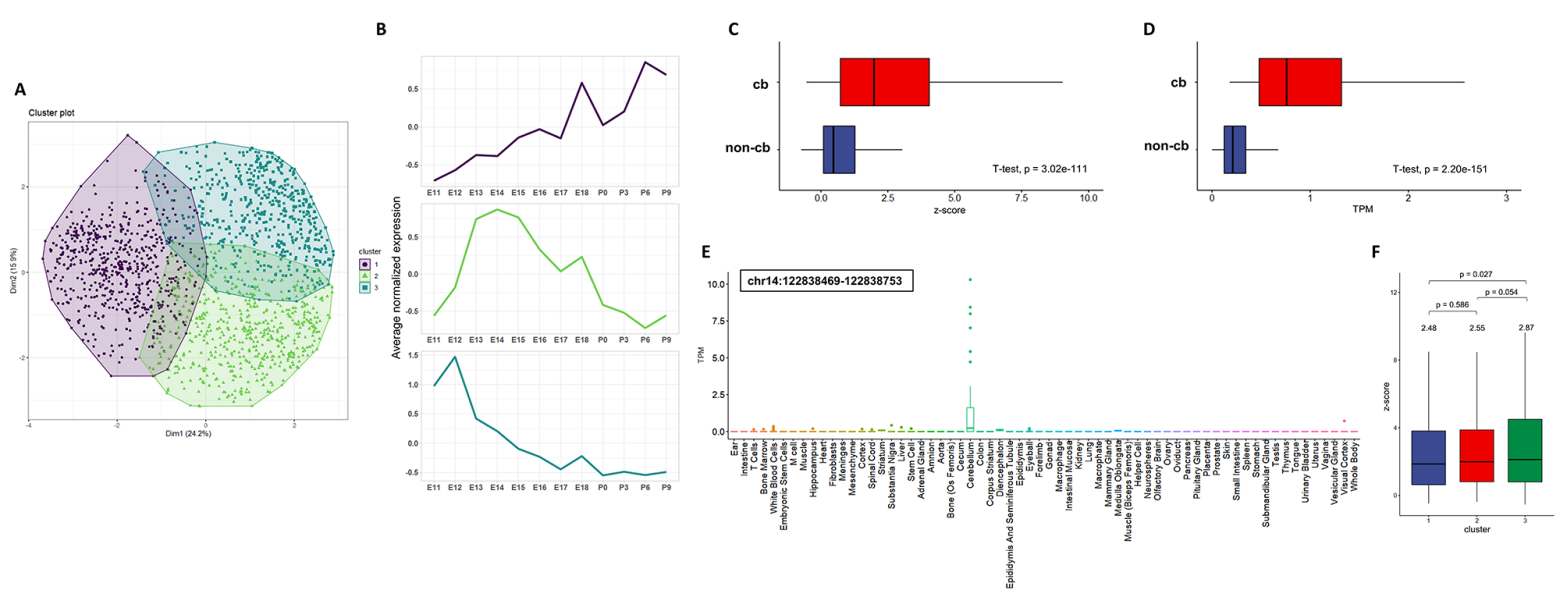
Robust cerebellar TE expression is dynamic throughout time and specific to cerebellar development. **(A)** Cluster plot of k-means analysis of robust cerebellar TEs. Three clusters are defined and the percentage of variance for dimensions 1 and 2 are 24.2% and 15.9% respectively. **(B)** Line plot showing the average z-score normalized expression values (average normalized expression) over time for Cluster 1 (top), 2 (middle) and 3 (bottom). **(C)** Box plot of maximum z-scores calculated for robust cerebellar TEs (cb) and the remaining TEs in the FANTOM5 mouse enhancer atlas (non-cb) from cerebellar samples. Z-scores were determined for all TEs using eRNA transcription across all mouse tissues submitted to the FANTOM5. A positive z-score in cerebellar samples indicated high eRNA expression in the cerebellum compared to the mean expression calculated for all tissues. **(D)** Box plot of the maximum expression level (TPM) out of all cerebellar samples for robust cerebellar TEs (cb) and the non-cerebellar TEs in the FANTOM5 mouse enhancer atlas (non-cb). **(E)** Boxplot showing eRNA expression of one tissue-specific robust cerebellar TE (chr14:122838469–122,838,753) for all FANTOM5 mouse tissues. The x-axis shows the various mouse tissues submitted to the FANTOM5 project and the y-axis represents normalized expression quantified at this TE in each tissue. **(F)** Box plot showing the maximum specificity z-scores for cerebellar samples for robust cerebellar TEs in each k-means cluster identified in our clustering analysis. P-values in this figure were generated using the t-test.

eRNA transcription from TEs has been found to be dynamic throughout development [4–6]. In a previous examination of cerebellar enhancer activity using post-translational histone modifications, we identified that enhancer activity is temporally specific, peaking during embryonic or postnatal stages [26]. However, the temporal activity of robust cerebellar TEs during brain development has yet to be assessed. With this in mind, we asked whether robust cerebellar TEs have dynamic activity throughout embryonic and early postnatal development. To explore this possibility, we conducted a k-means clustering of normalized eRNA expression patterns for robust cerebellar TEs to identify groups of co-expressed TEs. k-means analysis identified 3 co-expressed TE clusters, each peaking at three separate timepoints (Fig. 2A and B; Supplementary Table 3). We observed that Cluster 2 and 3 are active during embryonic development, peaking at E12 and E14, respectively; followed by declining expression over time. In contrast, the time point with the highest expression for Cluster 1 was found during postnatal development at P9 (Fig. 2B).

To verify whether eRNA expression is representative of enhancer activity in these clusters, we quantified H3K27ac signals at robust cerebellar TEs at E12, P0 and P9 using ChIP-seq data generated in a previous study. eRNA transcription was positively correlated, on average, with H3K27ac signal for cluster 1 (0.70), cluster 2 (0.94) and cluster 3 (0.97) (Supplementary Fig. 2A). K-means clustering analysis was also conducted for non-robust cerebellar TEs. Four clusters were identified with relatively similar patterns throughout time (Supplementary Fig. 2B). Clusters 1 and 4 peaked during postnatal stages, while clusters 2 and 3 peaked during late embryonic development (Supplementary Fig. 2C). Overall, this analysis indicates that cerebellar TEs are active during specific windows of development.

Previous examination of TE usage across human and mouse tissues identified that eRNA transcription is tissue-specific [3, 4, 6, 17, 30]. We asked whether this was also true for robust cerebellar TEs and assessed whether transcription from these elements was specific to the cerebellum compared to other mouse tissues. To do this, z-scores were calculated for each robust cerebellar TE using eRNA transcription across 64 mouse tissues submitted to the FANTOM5 [17]. A positive z-score in cerebellar samples indicated high eRNA expression in the cerebellum compared to the mean expression calculated for all tissues. Robust cerebellar TEs had a mean maximum z-score of 2.62 in cerebellar samples, which was significantly greater than the mean maximum z-score calculated for all other tissue samples (0.95, p-value = 3.02E-111) (Fig. 2C; Supplementary Table 4). We then assessed the maximum transcription levels (transcripts per million, TPM) for robust cerebellar TEs in cerebellar samples compared to other tissues. Expectedly, we found that maximum eRNA transcription was significantly higher on average in cerebellar samples (1.17 TPM) compared to other tissues (0.32 TPM, p-value = 2.20e-151) (Fig. 2D). Robust cerebellar TEs with the highest maximum z-scores exhibit high expression in cerebellar samples but minimal expression in other FANTOM5 tissues (Fig. 2E, Supplementary Fig. 3). Thus, robust cerebellar TEs exhibit tissue-specific eRNA expression and may be critical for fine-tuning the expression of genes in the developing cerebellum.

We then assessed tissue specificity across time, to evaluate whether tissue specific expression at robust cerebellar TEs was unique to a window of developmental time during cerebellar development. To do this, we calculated the average of the maximum z-scores for TEs in each of the k-means clusters. Mean z-score values for each cluster were 2.48, 2.55 and 2.87 for clusters 1, 2, and 3 respectively (Fig. 2F). We identified a significantly higher z-scores in cluster 3 when compared to cluster 1 (p-value = 0.027) indicating that specificity may arise during development. High average z-scores at each cluster, which peak at consecutive stages during development, indicates that robust cerebellar TE expression is specific to the cerebellum at embryonic and postnatal stages. Collectively, these results indicate that transcription at robust cerebellar TEs is specific to the cerebellum.

### TEs regulate genes important for functions specific to brain development

**Fig. 3:**
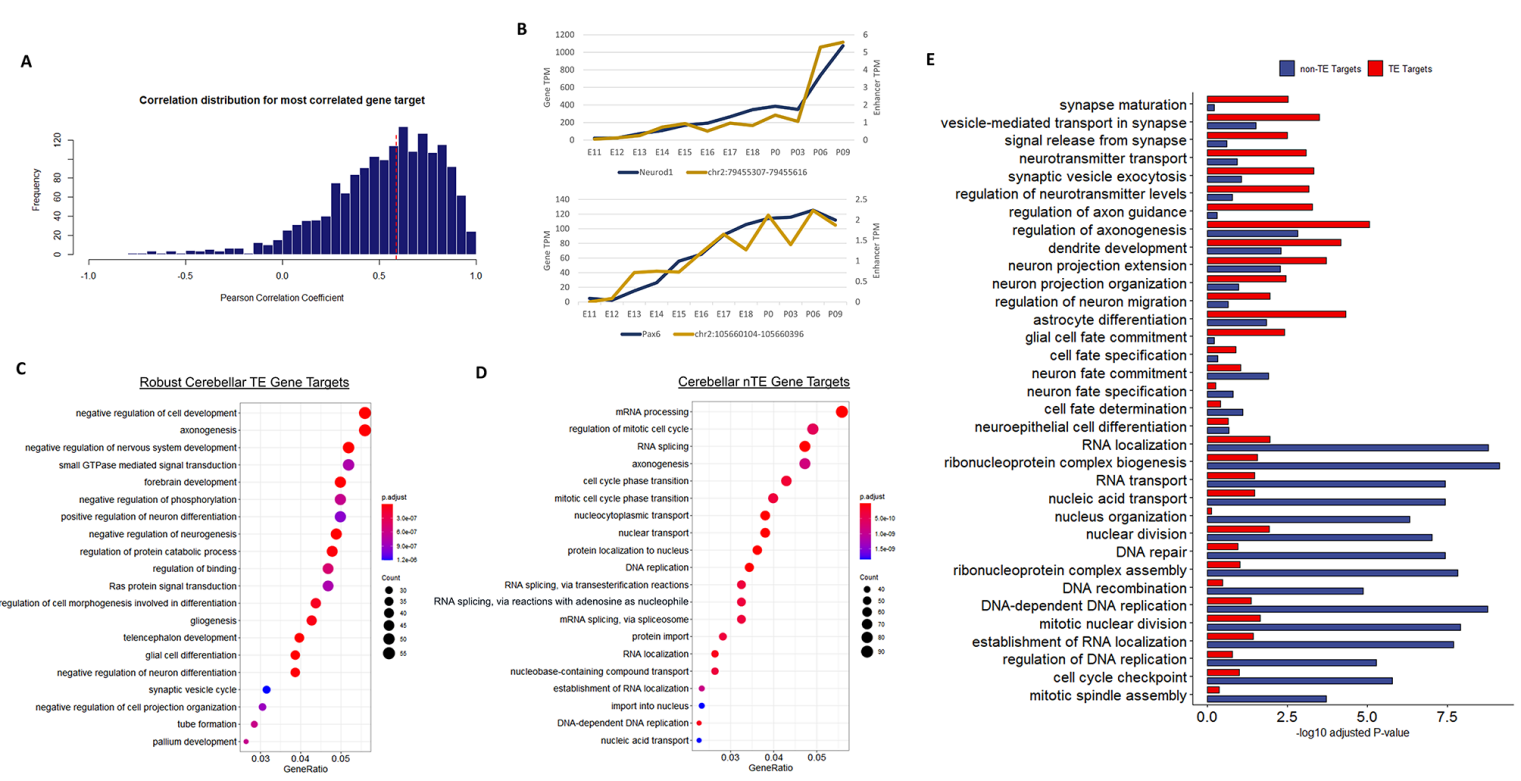
Molecular processes specific to brain development are regulated by robust cerebellar TEs. **(A)** Histogram showing the Pearson Correlation Coefficient of the most correlated gene target for all robust cerebellar TEs. Red line shows the cut off for significantly correlated gene targets (p-value < 0.05). P-values were determined using a two-tailed t-test. **(B)** Line plots of two representative TEs and their predicted target gene plotting TE eRNA expression (right y-axis) and target gene expression (left y-axis) throughout the cerebellar time course. TPM = transcripts per million. **C-D)** Gene ontology enrichment analysis for biological processes of robust cerebellar TE targets (C) and non-transcribed targets (D). Gene ratio, which is represented on the x-axis, is the ratio between the number of robust cerebellar target genes within a given GO term and the total number of target genes. The number of genes within that GO term (Count) is signified by the dot size and the adjusted p-value (p.adjust) is represented by dot color. **E)** Bar plot showing the enrichment of sorted GO terms for TE gene targets compared to non-transcribed enhancer gene targets. GO terms were categorized based on functions specific to the developing brain (ex. Synapse activity) and basic cell functions common to most developing tissues

To discover the molecular processes under TE regulation, we conducted a correlation analysis comparing eRNA and gene expression to identify potential TE target genes. We hypothesized that TEs regulate developmental processes specific to brain development as robust cerebellar TE expression was found to be cerebellum-specific. Our analysis consisted of two steps: First, the correlations between TE eRNA expression and expression of genes located in *cis* were calculated and second, the potential target genes were filtered for those located within the same conserved topological associating domain (TAD) [31]. Genes that were significantly correlated with eRNA expression (p-value < 0.05) were considered potential TE target genes. In total, we identified a positively correlated target gene (Pearson Correlation Coefficient > 0) for 89.4% (1488/1665) of TEs and a significantly correlated target gene for 45.1% (751/1665) of TEs (Fig. 3A, Supplementary Table 5). After using a cut off of a p-value < 0.05, significant TE-gene target pairs were highly correlated with a Pearson correlation co-efficient > = 0.62. To confirm that eRNA expression is indicative of enhancer activity, we calculated the correlation between eRNA transcription and H3K27ac signal at E12, P0 and P9 using a ChIP-seq dataset produced previously [26]. We found that eRNA expression and H3K27ac signal was positively correlated throughout time for the majority of robust cerebellar TEs with gene targets (583/751, 78%) with a mean Pearson correlation coefficient of 0.84.

In total, 964 genes were significantly correlated with a given TE. Of these genes, only 55/964 have previously been implicated in cerebellar development using a previously established database of genes critical for cerebellar development and function [32]. These included genes critical for cerebellar granule cell development such as Neurod1 and Pax6 [33, 34] (Fig. 3B). Strikingly, the vast majority of these targets (909/964) have not yet been investigated in the context of cerebellar development. The results of our analysis identify a rich resource of genes with novel regulatory roles in the developing cerebellum.

To identify potential molecular processes under TE regulation, Gene Ontology (GO) enrichment analysis was conducted for the 964 TE target genes. These results were compared to a GO enrichment analysis conducted for target genes of enhancers without eRNA expression. This list of genes was generated by subtracting TE target genes (964 genes) from target genes of cerebellar enhancers identified using H3K27ac and H34Kme1 peak signals alone; which results in a set of non-transcribed enhancer (nTE) target genes (1848 genes) [26]. TE targets genes were enriched for several biological processes specific to the developing brain such as “axonogenesis” (p-value = 5.29E-09), “glial cell differentiation” (p-value = 2.51E-09), “regulation of neuron differentiation” (p-value = 4.65E-05) and “neural precursor cell proliferation” (p-value = 2.32E-07) (Fig. 3C, Supplementary Table 6**)**. nTE target genes, on the other hand, were highly enriched for molecular processes involved in constitutive cell function, such as “mRNA processing” (p-value = 8.31E-17), “RNA splicing” (p-value = 9.31E-14), “mitotic nuclear division” (p-value = 1.65E-08), “DNA replication” (p-value = 2.84E-09) and “DNA repair” (p-value = 4.96E-08) (Fig. 3D**)**, Supplementary Table 7). Among the top 50 most significantly enriched GO terms for nTE targets, 82% (41/50) were constitutive molecular processes important for the development and function of all cell types.

To gain a more detailed understanding of this result, we plotted the enrichment (-log10-p-values) of biological processes specific to the brain for TE and nTE target genes. For comparison, we also plotted enrichment for constitutive cell functions. Compared to nTE targets, TE target genes were more highly enriched for brain-specific biological processes important for synapse development/function and neurite growth (Fig. 3E). nTE target genes showed a greater enrichment for constitutive biological processes than TE target genes. Furthermore, these findings were supported by a separate enrichment analysis of GO terms describing the compartment of the cell where the gene enacts its function. This latter analysis identified that the protein product of TE target genes can be found in neuronal-specific components such as “neuron to neuron synapse” (p-value = 2.90E-05) and “distal axon” (p-value = 8.06E-07) (Supplementary Fig. 4A, Supplementary Table 8**)**, while nTE targets can be found in the “nuclear envelope” (p-value = 9.79E-07), “spliceosome complex” (p-value = 1.88E-09) or at the “site of DNA damage” (p-value = 2.56E-05) (Supplementary Fig. 4B, Supplementary Table 9**)**. When considering GO terms focused on differentiation and specification, target genes of both types of enhancers show a similar level of enrichment (Fig. 3E). nTE target genes are also significantly enriched for “axonogenesis” (p-value = 3.18E-10) and “regulation of neurogenesis” (p-value = 3.18E-10) indicating that nTEs may regulate genes involved in neuron development, in addition to fundamental cellular processes. Collectively, this functional analysis indicates TEs drive gene expression programs required for neuron development while nTEs may regulate the expression of genes essential for constitutive cell functions as well as processes important for neuron differentiation.

To supplement our findings, we also identified putative target genes for non-robust cerebellar TEs (without histone marks present). We found a significantly correlated target gene for 2455/9322 cerebellar TEs potentially regulating 2744 genes (Supplementary Fig. 5A). When conducting a GO enrichment analysis, we found that these genes regulate processes critical for neuron development and maturation (Supplementary Fig. 5B). The majority of these putative targets overlapped with robust cerebellar target genes. We conclude that if these TEs can be verified to be active by other means, such as histone ChIP-seq or perturbation, it warrants investigation of their regulatory potential on cerebellar development.

### TEs regulate developmental processes at several stages of development

**Fig. 4:**
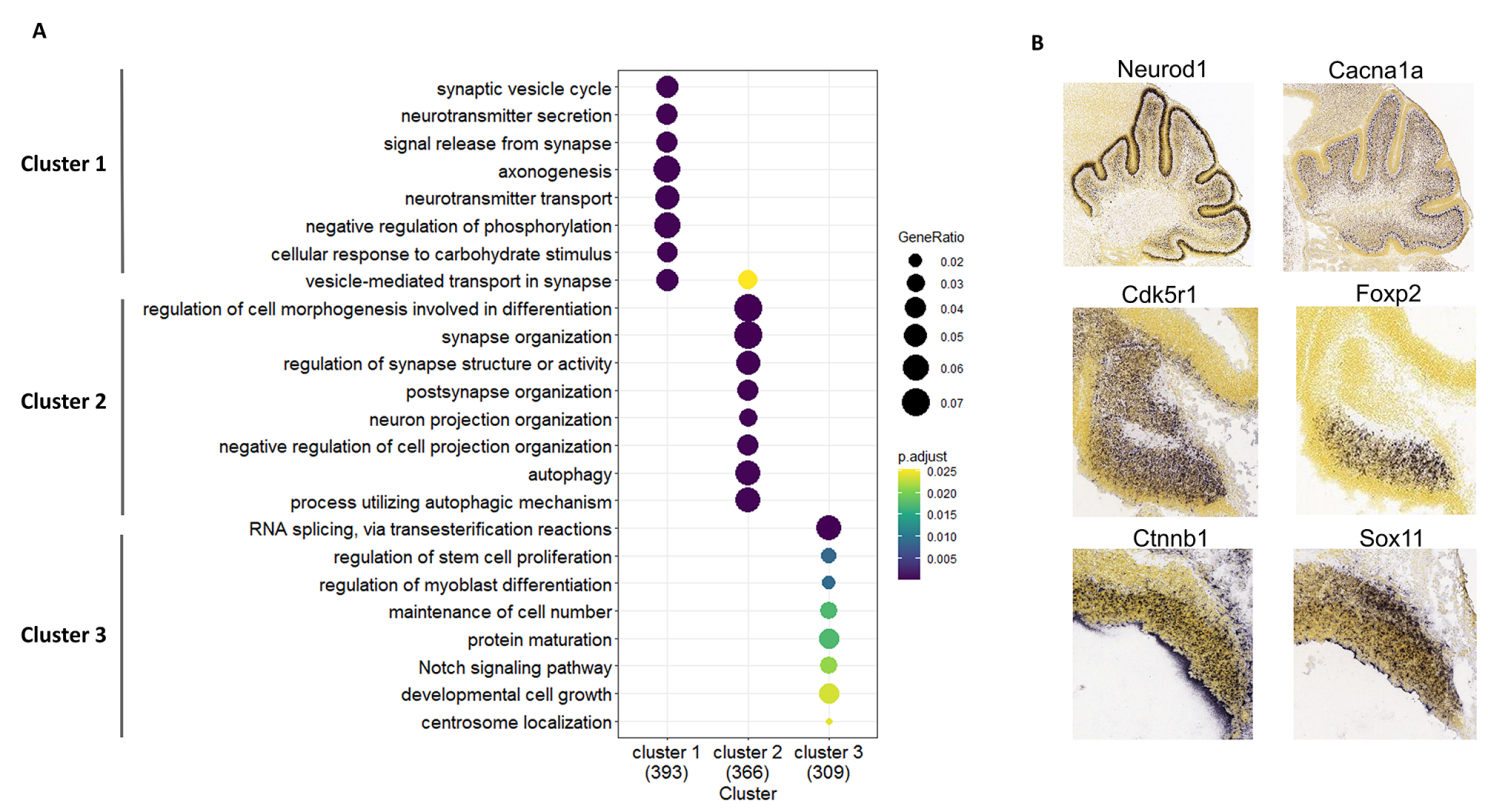
TEs with temporally specific expression are enriched for transient molecular processes typically occurring during embryonic or postnatal cerebellar development. **A)** A dot plot depicting GO enrichment analysis results analyzing target genes from each cluster. Dot color represents adjusted p-value and the dot size depicts the ratio of the number of TE target genes in a given GO category divided by the total number of genes analyzed. **B) **In situ hybridization images from the Developing Mouse Brain Atlas of cerebellar genes for each cluster, conducted at P4 (top), E15 (middle) and E11 (bottom)

We then asked whether TEs regulate genes driving biological processes occurring in temporally-specific time windows, identified by our k-means clustering analysis. First, we conducted a GO enrichment analysis of biological processes for target genes in each cluster. Each cluster was enriched for transient developmental events known to occur during the specific developmental stage with peak average expression (Fig. 4A, Supplementary Table 10). Cluster 1 target genes, which on average peaked in expression during postnatal stages, were enriched for “synaptic vesicle cycle” (p-value = 1.47E-04) and “neurotransmitter secretion” (p-value = 1.24E-07) and “neurotransmitter transport” (p-value = 2.07E-07), while Cluster 2 target genes, which on average peaked during mid-embryonic stages, were enriched for processes involved in the earlier stages of neuron differentiation, such as “neuron projection organization” (p-value = 1.76E-04) and synapse organization” (p-value = 1.90E-05). Cluster 3 target genes, which on average peaked in expression during early embryonic stages, were enriched for “maintenance of cell number” (p-value = 1.72E-02) and regulation of stem cell proliferation” (p-value = 8.65E-03).

Second, target genes with known cerebellar function were identified and their spatial expression pattern was examined using in situ hybridization (ISH) data from the Developing Mouse Atlas [35]. Overall, the results of the GO enrichment analysis were corroborated by the functions and expression patterns of known cerebellar genes within these clusters (Fig. 4B). In Cluster 1, we identified genes that are expressed in granule and Purkinje cells and are essential for the differentiation and maturation such as Neurod1 and Cacana1 [33, 34]. Cluster 2-contained genes, such as Foxp2 and Cdk5r1, which are expressed in cells within the cerebellar parenchyma which contain developing PCs and interneurons. Perturbation of the expression of these cerebellar genes results in aberrant development such as abnormal migration and deficits in dendrite growth [37, 38]. Cluster 3 contained genes expressed within the germinal zones of the cerebellum, such as Sox11 and Ctnnb1, important for neuronal precursor proliferation [39, 40]. Alteration of the expression of these genes results in a small cerebellum and abnormal neuronal differentiation. The functional analysis of target genes in each cluster demonstrates that TEs regulate processes important for distinct developmental stages during cerebellar development.

### Cerebellar TEs can regulate multiple gene targets and a subset of genes targets are regulated by multiple TEs

**Fig. 5:**
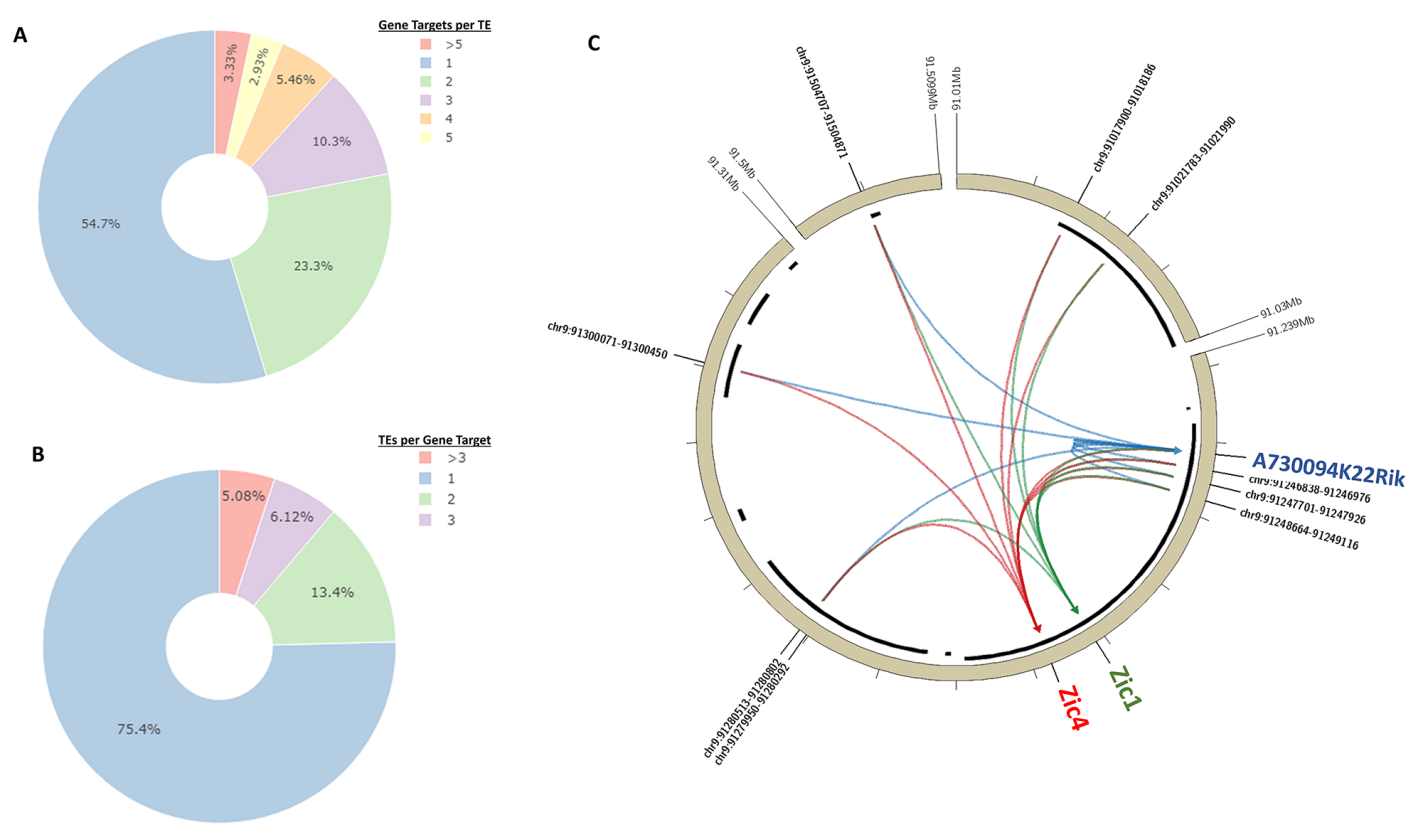
Robust cerebellar TEs regulate multiple target genes and target genes are regulated by multiple TEs. **(A)** Pie chart showing the proportion of TEs with one or more target genes. **(B)** Pie chart showing the proportion of target genes with one or more TEs. **(C)** An example of the complex and dynamic relationship of robust cerebellar TEs with their target genes at the Zic1/4 locus. A Circos plot showing the conserved topological associating domain (TAD) containing TEs (indicated by coordinates) predicted to regulate target genes Zic1, Zic4 and A730094K22Rik. Black bars indicate genomic locations with H3K27ac ChIP-seq peak signal. An arrow connecting a TE and a given gene indicates significant positive correlation between the eRNA expression at the TE and the target gene throughout cerebellar development

Transcription of eRNAs has previously been found to be associated with enhancers that regulate multiple target genes [40–43]. To determine whether robust cerebellar TEs have multiple putative gene targets, we examined the number of target genes per TE using the results of the TE target gene correlation analysis (Sect. 2). Interestingly, approximately half (44.32%) of robust cerebellar TEs were predicted to regulate two or more target genes (Fig. 5A).

These results prompted an examination of the number of robust cerebellar TEs regulating each predicted target gene as recent studies have shown that enhancers with redundant regulatory gene targets are a relatively common feature of the control of developmental gene expression [43–47]. We found that 24.6% of target genes were predicted to be regulated by 2 or more robust cerebellar TEs (Fig. 5B). When considering TEs in different k-means clusters, we found that most TEs that target the same gene were found to be in the same cluster (74%), while 23% of TEs with the same target were found in 2 different clusters and 3% were found in 3 clusters (Supplementary Fig. 6A). For the 26% of TEs from different clusters with the same putative target, we found they are distributed 318 kb from each other, on average. This indicates that TEs can be distributed in several distal locations within a TAD, despite regulating the same gene (Supplementary Fig. 6B).

Target genes with multiple TEs were then assessed for their association with cerebellar development. Out of the 321 target genes with more than one TE, 27 (8.4%) of these genes were previously associated with cerebellar development. The 27 genes make up 38% (27/71) of the total number of target genes annotated to have a cerebellar phenotype when perturbed. This indicates that a subset of genes critical for cerebellar development may rely on multiple regulatory elements for proper expression. For example, 8 highly correlated robust cerebellar TEs were identified in the same conserved TAD as Zic1 and Zic4, all of which were predicted to regulate both genes (Fig. 5C). Three out of the 8 robust cerebellar TEs may also regulate a gene not yet associated with cerebellar development, i.e. A73009K22Rik. In contrast, Zic1 and Zic4 have previously been found to control the development of glutamatergic cell types in the cerebellum and combined, heterozygous loss of function mutations of these genes have been associated with Dandy-Walker Syndrome [48]. Taken together, an expanded examination of TEs and their potential target genes has revealed that TEs may regulate multiple target genes, acting as drivers of gene expression programs critical for development.

### TE eRNA transcription occurs in the same cells as their predicted target genes

Regulatory relationships between enhancers and their potential target genes have previously been implicated using reporter assays where reporter signal and gene expression in the same regions suggest a regulatory relationship between enhancers and putative target genes [49]. In this study, we compared the spatial expression pattern of eRNA transcripts expressed from TEs, as proxy for enhancer activity, with the spatial expression pattern of a predicted target gene. Co-expression within the same population of cells would favor a regulatory relationship.

**Fig. 6:**
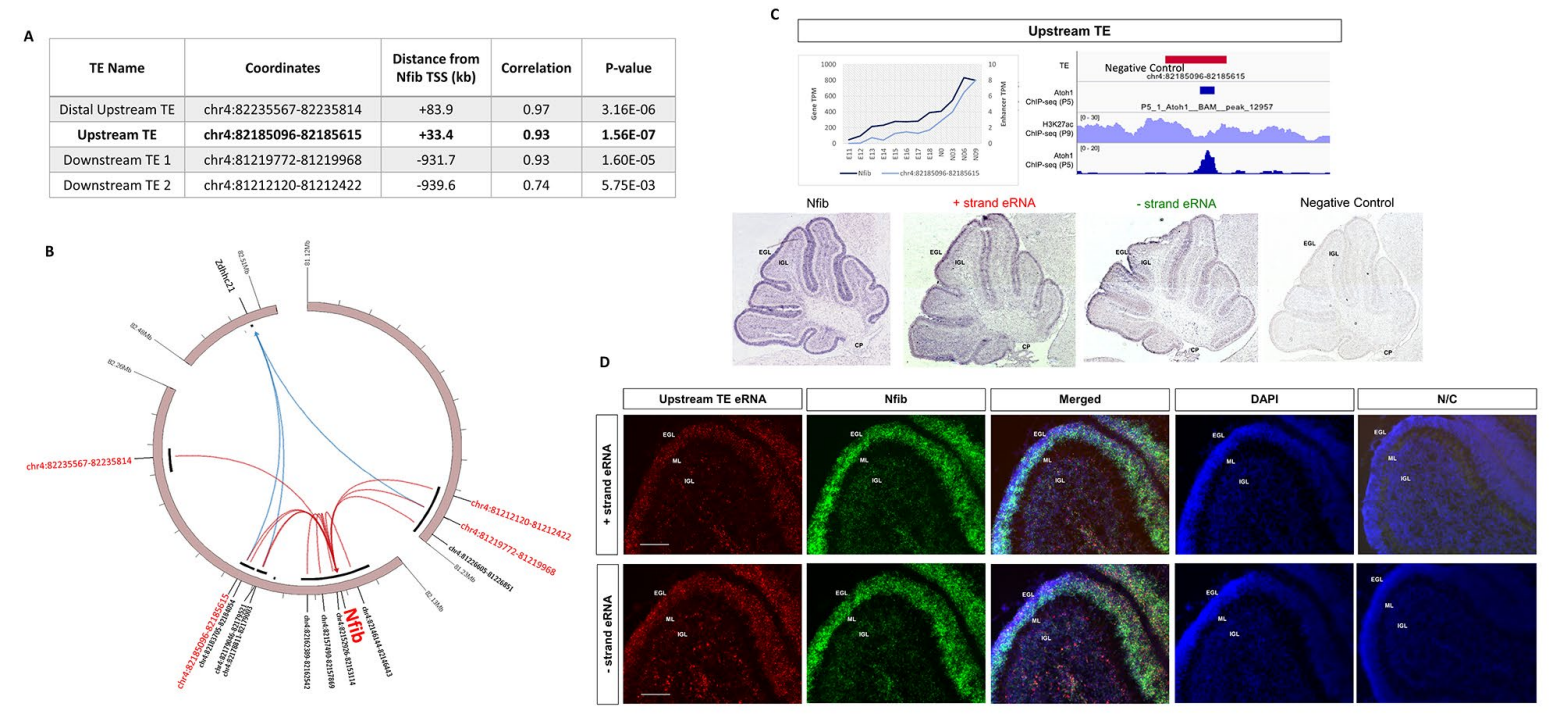
TEs predicted to regulate Nfib expression in the developing cerebellum. **(A)** A table containing the Atoh1-bound robust cerebellar TEs predicted to regulate Nfib. Columns show the coordinates of each TE in the mouse genome (mm9), the distance from the Nfib transcriptional start site (TSS), and Pearson Correlation Coefficient calculated for TE and Nfib expression. **(B)** A Circos plot of the conserved TAD containing TEs (indicated by coordinates) predicted to regulate target genes Nfib and Zdhhc21. Black bars indicate genomic locations with H3K27ac ChIP-seq peak signal. An arrow connecting a TE and a given gene indicates significant positive correlation between the eRNA expression at the TE and the target gene throughout cerebellar development **C)** Spatial eRNA expression analysis for Nfib and Upstream TE. Upper left panel: Expression pattern (TPM) for Nfib and TE throughout cerebellar development. Upper right panel: IGV Browser tracks showing transcribed enhancer (TE) location, Upstream TE H3K27ac ChIP-seq signal at P9 and Atoh1 ChIP-seq peak location and signal at P5. Lower panel: ISH at P6 for Nfib, Upstream TE “-“ strand eRNA and “+” strand eRNA. EGL: External granule layer, IGL: Inner granule layer, CP: choroid plexus. **D)** RNAscope fluorescent in situ hybridization of Nfib (green), Upstream TE + and – strand (red) eRNAs, DAPI (blue) and negative control (N/C). Merged images show all channels overlaid. Stains were conducted on P6 mouse cerebella. EGL: External granule layer, ML: Molecular layer, IGL: Inner granule layer, CP: choroid plexus. Scalebar: 100 μm

To identify TEs regulating a gene critical for postnatal granule cell development for biological validation, we first isolated TEs and target genes from Cluster 1 derived from our k-means clustering analysis, which peaked in expression during postnatal development (Fig. 4A). Cluster 1 TEs were then filtered based on putative target gene function, where genes that have previously been implicated in postnatal granule cell development were prioritized; which was determined using the Cerebellar Gene Database. The remaining TEs were then filtered for elements potentially active in granule cells by identifying TEs bound by Atoh1, the lineage defining molecule for glutamatergic cells in the developing cerebellum [50]. This was determined using an Atoh1-ChIP-seq dataset conducted previously in the postnatal cerebellum [51]. The resulting list consisted of 142 putative target genes regulated by 80 TEs. Target genes were sorted by the number of Atoh1-bound TEs. Nfib, a key regulator of postnatal granule cell differentiation [52], was the significant putative target gene with the most, four, Atoh1-bound robust cerebellar TEs and was chosen for validation (Fig. 6A). eRNA expression from two of the four Nfib TEs were also significantly correlated with Zdhhc21 expression, a gene found within the same TAD as Nfib. Zdhhc21 has not been previously associated with the development of the cerebellum. TEs predicted to regulate Nfib were found at various distances from the Nfib TSS, with two located upstream (labelled Distal Upstream TE and Upstream TE ) and two located downstream (labelled Downstream TE 1 and 2) (Fig. 6B). To visualize the bi-directional expression pattern for each eRNA we performed a standard colorimetric in situ hybridization (ISH) experiment on sections taken from the postnatal cerebellum (P6). We constructed ISH probes for eRNAs transcribed from the 5’ end (- strand eRNA) and 3’ end (+ strand eRNA) of the TE (See Methods, Supplementary Fig. 7A).

PCR probes were successfully amplified for eRNAs from 3/4 TEs (transcripts from the Downstream TE 2 were not detected). ISH was also conducted for Nfib, in addition to the – and + strand eRNAs for each TE, to evaluate expression patterns and a potential regulatory relationship. Nfib expression was identified in the external granule layer (EGL) and the internal granule layer (IGL) similar to previous observations [53]. These two regions contain proliferating and differentiating granule cells. Strikingly, we observed a similar expression pattern for the – and + strand Nfib TE eRNAs, observing expression in the EGL and IGL (Fig. 6C, Supplementary Fig. 7B,C). This result indicated that TEs predicted to regulate Nfib may be active in the same regions as Nfib.

We then conducted RNAscope to gain a higher-resolution depiction of eRNA transcription. This technique also allows the detection of TE eRNA and Nfib transcription in the same tissue section using two separate fluorescent dyes. Probes were constructed for + and – strand eRNAs transcribed from the Upstream TE as well as for Nfib and cerebellar sections from P6 were stained. We observed expression of the Upstream TE + and – strand eRNAs in the EGL and the IGL (Fig. 6D). Co-localization between Nfib and both Upstream TE + and – strand eRNAs was identified within the cells of the EGL and the IGL. The detection of Upstream TE and Nfib transcripts in the same cells indicates that this robust cerebellar TE is active within developing postnatal granule cells. Additionally, our findings using RNAscope mirrored the results of the standard colorimetric ISH. This analysis also validates our gene target prediction analysis, identifying a possible regulatory relationship between Nfib and the Upstream TE.

### Online dataset resource

The findings from our study can be explored and exported through an online resource https://goldowitzlab.shinyapps.io/trans-enh-app/. This resource also provides links to relevant databases (ex. Mouse Genome Informatics, FANTOM5 Data Portal, Allen Brain Atlas) for further investigation of TE and putative target gene expression and function.

## Discussion

Transcribed enhancers (TEs) are a subset of enhancer elements that are important for temporal- and tissue-specific gene expression underlying development [16]. Functionally annotating these regulatory regions is an important step in unraveling the complex network controlling the development of the numerous cell-types in the brain. To this end, we previously documented that enhancer elements active during cerebellar development, identified through profiling enhancer-associated histone modifications, have temporally and spatially specific activity and regulate developmental processes occurring specifically during embryonic or postnatal development [26]. However, the role TEs play in the context of embryonic and postnatal cerebellar development has not been explored. In this work, quantification of eRNA transcription in the cerebellum leads to the discovery of the first catalog of robust cerebellar TEs active during embryonic and early postnatal development. Robust cerebellar TEs exhibit temporally specific transcription peaking during embryonic or postnatal stages and regulate genes involved in molecular processes specific to brain development. We then validate our findings by using FISH to identify eRNA expression as a proxy for enhancer activity in the same cells as the putative target gene.

### eRNA is a signal of enhancer activity and is correlated with target gene expression in the developing cerebellum

Understanding the functional relevance of enhancers active during development requires assigning active enhancers to their downstream target genes. We identified putative regulatory targets by identifying genes with expression significantly correlated with robust cerebellar TE transcription throughout cerebellar development. This analysis capitalizes on previous observations of a strong correlation between eRNA transcript and neighboring mRNA transcription during cellular differentiation and/or activation [4, 17]. eRNA transcription is also highly correlated with enhancer-associated histone modifications and physical interaction between enhancers and the promoters of target genes. Other studies have found a tight relationship between enhancer transcription and transcription factor activity [54], as well as enhancer and promoter function [5]. These findings demonstrate that eRNA levels can be a useful measure of enhancer activity and emphasize the advantages of using transcription as a predictor of target gene regulation. Our study is the first, to our knowledge, to apply this approach to an in vivo transcriptomic time course of cerebellar development to gain insight on the role of TEs during embryonic and postnatal cerebellar development. Additionally, we validate enhancer activity in the developing cerebellum by conducting fluorescent in situ hybridization of TE eRNA expression. TE activity has only been previously demonstrated using this technique in cultured neurons and cancer cell lines [24, 55]. However, we apply this technique to fixed postnatal mouse brain tissues, in order to detail spatial eRNA expression in vivo. Co-localization of eRNAs expressed from a robust cerebellar TE with Nfib expression, a gene essential for cerebellar granule cell development, confirms expression within granule cells and implies a potential regulatory relationship. This observation, importantly, also serves as partial validation of our enhancer-gene target prediction analysis.

### TEs regulate functions important for neuron development with temporal specificity

An examination of eRNA expression during cerebellar development has revealed that robust cerebellar TEs exhibit temporally-specific patterns of expression. The identification of the putative target genes of robust cerebellar TEs revealed that TEs regulate transient and temporally specific developmental events in the cerebellum. During early embryonic stages, TEs regulate processes critical for neural progenitors such as proliferation and maintenance of cell number. However, as development progresses there is a shift in usage to TE’s driving the expression of genes important for the early stages of neuron differentiation, such as neuron migration. Indeed, similar shifts in chromatin and enhancer activity has been observed in the developing cerebellum and developing forebrain using the analysis of histone modifications and DNase hypersensitive sites [6, 56, 57]. Previous studies have found that TEs and their respective eRNAs regulate genes critical for differentiation of various cell-types such as skeletal myoblasts [18, 19], osteoclasts [20], T-cells and B-cells [15, 21], cardiomyocytes [22] and embryonic stem cells [17]. For example, Mousavi et al. (2013) identified eRNA transcription at previously characterized enhancers that regulate genes critical for myogenic differentiation. Interestingly, the depletion of these eRNAs resulted in reduced chromatin accessibility and RNAPII occupancy at the MYOD1 locus and perturbed myogenic differentiation [18]. With the present findings, the stage is set to validate the regulatory relationship between cerebellar TEs, their putative target genes and the impact on neuronal differentiation.

Robust cerebellar TE target genes were enriched for functions specific to the brain, which are typically seen during later stages of embryonic and during early postnatal development. These included developmental events critical for neuron maturation and connectivity such as neurite growth and synapse activity/organization. Recently, Tuvikene et al. (2021) identified an evolutionarily conserved intronic TE regulating the expression of Bdnf, a protein critical for the maturation of synaptic connections and regulation of synaptic plasticity [58]. The binding of various activity-dependent TFs to this intronic TE, as a result of BDNF-TrkB signaling (in reporter and endogenous assays), confirmed that this TE participates in BDNF-TrkB signaling and neuronal-activity-induced expression of Bdnf. In addition to neuronal maturation, TEs are involved in the response to neuronal activity [3, 24, 59, 60]. This indicates that TE gene expression regulation is important not only during development but in driving the transcriptional response to signal dependent activity in mature neurons. Our analysis of TE target gene function reveals that TEs likely regulate the expression of genes involved in neuronal differentiation and maturation in the developing cerebellum.

GO term enrichment results were compared for TE and nTE target genes, highlighting that robust cerebellar TEs are more enriched for neuron-specific functions while non-transcribed enhancers are enriched for constitutive cell functions. We speculate that since non-transcribed cerebellar enhancers regulate fundamental cellular processes, these elements may be utilized across many tissue types. Enhancers with ubiquitous activity across tissues have previously been described [4, 30]. Ubiquitous enhancers are highly conserved and are more likely to overlap with CpG islands. More importantly, these enhancers are predicted to regulate genes involved in constitutive cell processes utilized by most cells. Further evaluation of non-transcribed enhancer activity across tissues may identify whether these elements are ubiquitous enhancers.

### Genes critical for cerebellar development are regulated by multiple TEs

Enhancers that regulate a common target gene and display a partially overlapping spatial and temporal activity are known as ‘shadow enhancers’ [44]. Shadow enhancers are a mechanism of redundancy that helps improve the precision of gene expression and provides phenotypic robustness during development under conditions of genetic or physiological stress. We identified that a subset of target genes is regulated by multiple enhancers indicating that shadow enhancers are a common regulatory mechanism for genes critical for cerebellum development. Similar observations have been found in other developing tissues, as recent evidence shows that shadow enhancers are highly prevalent in the genome and regulate genes critical for development [45, 61, 62]. A study by FANTOM5 found that enhancer redundancy is common in the human genome by identifying that approximately 80% of the 2,206 TE target genes with developmental function were associated with two or more co-transcribed enhancers [4]. Previous studies have shown that shadow enhancers are important for brain development in the context of neurogenesis, neuronal activity and neurodevelopmental disorders [47, 61, 63–67]. Dickel et al. (2018), investigated the impact of deleting functionally redundant ultra-conserved enhancers predicted to regulate Arx in the developing telencephalon, a gene important for brain development; mutations in which cause a variety of severe neurological phenotypes [68]. Mouse lines with single enhancer deletions exhibited only a slight reduction in Arx expression, however this still resulted in a decrease in striatal cholinergic neurons and neocortical GABAergic interneurons. Interestingly, pairwise enhancer deletions exacerbated these phenotypes, resulting in a greater reduction of Arx expression and almost complete loss of striatal cholinergic neurons and a further decrease in the density of interneurons [61]. Collectively, evidence from both single-enhancer and genome-wide enhancer perturbation studies have shown the importance of enhancer redundancy in development. Our findings indicate that shadow enhancers are important for gene expression regulation during embryonic and early postnatal cerebellar development.

## Conclusions

Our study identifies a role for TEs in the context of the developing brain. Our datasets serve as a valuable resource for future studies that will further characterize the relationship between these TEs and their target genes. The results of our study also narrow the search for functionally-associated sequences important for cerebellar development, but also reveals a larger set of genes that have thus far been uninvestigated in the developing cerebellum. These genes could serve as entrees to perturbation studies to appreciate their role in development and neurodevelopmental disorders. To facilitate these efforts, we have made the results of our analyses available as an online resource: https://goldowitzlab.shinyapps.io/trans-enh-app/. This allows the research community to efficiently explore, curate and export our data for future studies.

## Materials and methods

All methods were carried out in accordance with relevant guidelines. The results in this study are reported in accordance with ARRIVE guidelines.

### eRNA quantification of FANTOM5 transcribed enhancer atlas

Bidirectional eRNA transcription was quantified for 44,259 mouse transcribed enhancers identified previously by the FANTOM5 Consortium [4, 17]. Briefly, TEs were identified as loci with non-overlapping bi-directional transcription by merging pairs of divergent TSS clusters separated by at most 400 bp. For each bidirectional locus, strand-wise expression was quantified in windows of 200 bp immediately flanking its derived midpoint. Enhancers were predicted from loci that had CAGE tags supporting expression on both strands (in both windows) in at least one CAGE library, at most 80% of supporting CAGE tags from pooled CAGE libraries falling into one strand window (directionality), and a greater fraction of plus strand tags than minus strand tags from pooled CAGE libraries in the window describing plus strand expression and vice versa. Bidirectional loci were then filtered to be distant to TSSs (500 bp) and exons (200 bp) of annotated protein-coding and multi-exonic non-coding genes.

### Identification of TEs expressing eRNA in the developing cerebellum

To identify TEs active in the developing cerebellum, whole mouse cerebella samples were collected from 12 time points across cerebellar development (embryonic days 11–18 at 24 h intervals and every 72 h until postnatal day 9) [28]. RNA was isolated and subjected to Cap Analysis of Gene Expression followed by sequencing (CAGE-seq) as described [28]. eRNA transcription was then quantified for the FANTOM5 TE atlas [4, 17]. To mine this dataset in which FANTOM5 TEs express eRNA in the developing cerebellum, bioinformatic filtering was conducted based on criteria adapted from Yao et al. (2015) [25]. The criteria included (1) a conservative expression cut-off of > 0.5 transcripts per million (TPM), (2) expression detected during at least three time points throughout the time course and (3) expression found in at least 2/3 biological replicates for a given time point. These criteria were chosen to include enhancers with replicable eRNA expression which is considered strong evidence of transcriptional activity and to rule out artefactual signals such as genomic DNA contamination.

### Comparison to cerebellar histone ChIP-seq datasets

Histone ChIP-seq and peak determination for H3K27ac and H3K4me1 was previously conducted for cerebella collected at E12, P0 and P9 [26]. This dataset was downloaded from Gene Expression Omnibus (GEO) (GSE183697). Co-ordinates of peak signal for both marks were intersected with TEs expressing eRNAs in the developing cerebellum using Bedtools v.2.28 [69]. TEs intersecting with both marks were considered robust cerebellar TEs. For the comparative analysis with cerebellar postnatal enhancers previously published by Frank et al. (2015), H3K27ac and DNase-seq peak coordinates were downloaded from GEO (GSE60731). For the comparisons, these sequences were overlapped with cerebellar TEs and robust cerebellar TEs identified using P9 H3K27ac ChIP-seq peaks using Bedtools v.2.28 [69].

### Gene Target Prediction Analysis

Candidate gene targets for robust cerebellar TEs were identified through a sequential approach, including a correlation analysis of expression and filtering by genomic location. First, to identify gene targets with similar expression patterns throughout developmental time, correlations were calculated for each robust cerebellar TE and all genes located using expression from all 12-time points in the cerebellar time course [28] (Fig. 1). Second, potential gene targets were filtered for those with statistically significant positive correlation with TE eRNA transcription (r > 0, calculated using t-tests, adjusted P-value < 0.05). Third, highly correlated gene targets were then filtered based upon location within the same conserved topological associating domain (TAD) as identified previously [31]. These TADs are conserved between different cell types and even across species and were established using Hi-C data generated from mouse embryonic stem cells.

### Gene Ontology Enrichment Analysis

Gene ontology (GO) enrichment analysis was conducted using clusterProfiler [70]. TE gene targets were used as input for this analysis. To construct the list of targets of non-transcribed enhancers, TE targets were subtracted from the list of target genes of enhancers active the developing cerebellum, based on histone ChIP-seq profiles, which was determined previously [26]. GO enrichment was conducted for Biological Processes and Cellular Component GO terms. Biological Processes are “the larger processes, or ‘biological programs’ accomplished by multiple molecular activities” [71] while Cellular Component consists of GO terms describing “the locations relative to cellular structures in which a gene product performs a function, either cellular compartments (e.g., mitochondrion), or stable macromolecular complexes of which they are parts (e.g., the ribosome)” [71].

### k-means clustering analysis

For robust cerebellar TEs, kmeans clustering of eRNA expression patterns was conducted. Input for this analysis was Z-score normalized eRNA expression for robust cerebellar TEs including all 12 developmental timepoints. k-means clustering is an unsupervised learning approach that was used to group TEs according to their activity profile. This heuristic algorithm uses the centroid principle which is the geometric center of a cluster and will minimize the distance between a point and a centroid to assign this point to a cluster. With this approach it is necessary to define the number k, and therefore the cluster number that we will attribute to our data. The k value (number of clusters) was determined using an Elbow analysis (k = 3). These k-means clusters were validated by calculating the Pearson correlation coefficient between the points belonging to the same group.

### Data Analysis

All plots and statistical analyses were generated in R version 3.2.3 (R Core Team, 2014) and figures were produced using the package ggplot2. Bedtools v2.29.1 [69] was used for comparing and overlapping the genomic coordinates of peaks and existing genomic features described in the manuscript. Boxplots represent the mean (centre line), first and third quartiles (top and bottom of box, respectively) and confidence intervals (95%; black lines). Genome browser screenshots were taken from the Integrated Genomics Viewer (IGV) genome browser [72].

### Mouse strains and husbandry

C57BL/6 J mice, originally purchased from JAX laboratory, were maintained and bred in our pathogen-free animal facility with 12/12 hour light/dark cycle and a controlled environment. Embryonic ages utilized in these experiments were confirmed based upon the appearance of a vaginal plug. The morning that a vaginal plug was detected was designated as E0.5. Pregnant females were cervically dislocated and embryos were harvested from the uterus. Postnatal ages were determined based upon the date of birth with the morning of the observation of newborn pups considered as P0.5. All studies were conducted according to the protocols (protocol ID: A20-0164-R002) approved by the Institutional Animal Care and Use Committee and the Canadian Council on Animal Care at the University of British Columbia.

### Tissue Preparation for Histology

C57BL/6 J mice (male and female) at P3.5, P6.5 and P9.5 were perfused through the heart with a saline solution followed by 4% paraformaldehyde/0.1 M PBS. The brain was isolated and further fixed in 4% paraformaldehyde in 0.1 m PB for 1 h at room temperature. Fixed tissues were rinsed with PBS, followed by cryoprotection with 30% sucrose/PBS overnight at 4 °C before embedding in the Optimal Cutting Temperature compound for sectioning. Tissues were sectioned at 12 μm for in situ hybridization and immunofluorescence experiments and cryosections were mounted on Superfrost slides (Thermo Fisher Scientific), air dried at room temperature, and stored at − 80 °C until used. Sagittal sections were cut from one side of the cerebellum to the other (left to right, or vice versa).

### In situ hybridization for the detection of Nfib and Nfib TEs

Probe design for eRNAs and Nfib: A cDNA library was synthesized from RNA isolated from C57BL6/J P9.5 mouse cerebella using a cDNA synthesis kit (Invitrogen) from RNA. A cDNA PCR amplicon corresponding to Nfib and each eRNA was produced from this cDNA library, using forward and reverse primers specific to each eRNA (Supplementary Table 11). For eRNAs, CAGE-seq analysis of TEs identified transcriptional start sites of respective eRNAs but not the termination site. To identify primer pairs to amplify a cDNA fragment of the eRNAs to generate probes for ISH, tiling PCR experiments were performed. For a given eRNA, the first primer designed just downstream of the identified eRNA TSS identified by CAGE-seq. The second primers were then designed approximately every 100 bp downstream of the TSS up to 1 kb. PCR was run for every primer pair and the longest amplicon indicated the approximate size of the eRNA. This primer pair was then used for probe generation for ISH. Sense and antisense riboprobes corresponding to the amplified cDNA fragment were synthesized and labeled with digoxygenin (DIG)-UTP. For RNAscope, the genomic locations and sequences of Nfib and Nfib eRNAs were sent to Bio-techne ACD to generate probes for hybridization (Supplementary Table 12).

Standard colorimetric detection: cDNA fragments amplified from Nfib eRNAs were cloned into the pGEM-T vector (A3600, Promega) for the generation of cDNA templates. cDNA templates for the sense and antisense riboprobes is specifically made using the primers M13F: GTTTTCCCAGTCACGAC or M13R: CAGGAAACAGCTATGAC and the eRNA-specific forward or reverse primers. Riboprobes are produced using SP6 or T7 RNA polymerase (#EP0133 and #EP0111, Thermoscientific, respectively) with the corresponding cDNA templates. The riboprobes were then precipitated using 5 M ammonium acetate and 100% EtOH in RNase-free environment. Riboprobes were denatured at 72 °C for 10 min, and incubated on ice for 5 min, then mixed with ULTRAhyb hybridization buffer (AM8670, Applied Biosystems) preheated at 68 °C. Prior to hybridization, sections were acetylated with acetic anhydride in 0.1 M triethanolamine at pH 8.0 and dehydrated with graded concentrations of RNase-free ethanol. Sections prepared for histology (see below) were first incubated with ULTRAhyb hybridization buffer at 68 °C in a humid chamber for 30 min, then replaced with riboprobe in ULTRAhyb hybridization buffer at 68 °C overnight. After hybridization, the slides were rinsed with descending concentrations of salt: 4x SSC, 2x SSC, 1x SSC and 0.5x SSC at 55 °C, and then incubated with an anti-Dig antibody (11,093,274,910, Roche) for 2 h at room temperature. The slides were washed with maleic buffer, followed by reaction buffer, then the slides were colorized with NTP/BCIP (11,681,451,001, Roche). Following colorization, the slides were rinsed with 0.1 M PB, then post-fixed in 4% paraformaldehyde, and washed with distilled water. The slides were dehydrated with graded concentrations of ethanol and xylenes. Glass coverslips were applied to the slide using Paramount (SP15-500, Fisher Scientific).

RNAscope fluorescent dye detection: To look at RNA level expression of Nfib and eRNAs simultaneously and at higher resolution, Bio-techne ACD’s RNAscope Multiplex Fluorescent V2 Assay kit (single molecule RNA fluorescent in situ hybridization) was used according to manufacturer’s instructions. The RNAscope technology uses tyramide signal amplification which suppresses background and boosts the signal such that individual RNA molecules can be detected as puncta - The “ZZ” probe design only allows amplification to build upon consecutively bound probes on the target, thereby ensuring that each puncta represents only real signal [73]. Briefly, the slides were post-fixed in 4% PFA for 30 min, dehydrated in graded ethanol solutions and permeabilized with a protease treatment for 15 min. Slides were then hybridized with the probes overnight at 40 °C. After this, the signal amplification tree was built by sequentially incubating slides in Amplifiers 1,2 and 3 at 40 °C. The first amplification strand, Amplifier 1, only hybridizes to the “ZZ” s. This was followed by developing the fluorescent channels that involved incubation with HRP attached to the channel-specific sequence, adding the fluorescent dye, and then adding HRP blockers so the other channels can be developed similarly. All these incubations were done at 40 °C for durations based on the user manual guide provided by the manufacturer. After the final HRP blocking step, slides were incubated in DAPI to counterstain for 5 min before cover slipping with FluorSave mounting medium.

### Image analysis and microscopy

Analysis and photomicroscopy were performed using a Zeiss Axiovert 200 M microscope with the Axiocam/Axiovision hardware-software components (Carl Zeiss) and downstream image analysis was conducted using the AxioVision software v.4.9.1 (Carl Zeiss). For all results, observations were based on a minimum of 3 embryos per experiment.

## Electronic supplementary material

Below is the link to the electronic supplementary material.

Supplementary Material

Supplementary Material 2

Supplementary Material 3

Supplementary Material 4

Supplementary Material 5

Supplementary Material6

Supplementary Material 7

Supplementary Material 8

Supplementary Material 9

Supplementary Material 10

Supplementary Material 11

Supplementary Material 12

Supplementary Material 13

Supplementary Material 14

Supplementary Material 15

Supplementary Material 16

Supplementary Material 17

Supplementary Material 18

Supplementary Material 19

Supplementary Material 20

## Data Availability

The CAGE-seq datasets generated in this study is available online through the FANTOM5 data portal: https://fantom.gsc.riken.jp/data/. Histone ChIP-seq and peak determination for H3K27ac and H3K4me1 was previously conducted for cerebella collected at E12, P0 and P9 (Ramirez et al., 2021). This dataset was downloaded from Gene Expression Omnibus (GEO) (GSE183697). For the comparative analysis with cerebellar postnatal enhancers previously published by Frank et al. (2015), H3K27ac and DNase-seq peak coordinates were downloaded from GEO (GSE60731).
