# Supplementary figures and images for: Identification and characterization of transcribed enhancers during cerebellar development through enhancer RNA analysis
Source: BMC Genomics. 2023 Jun 26;24:351. doi: 10.1186/s12864-023-09368-4 (PMC10291752; doi:10.1186/s12864-023-09368-4)

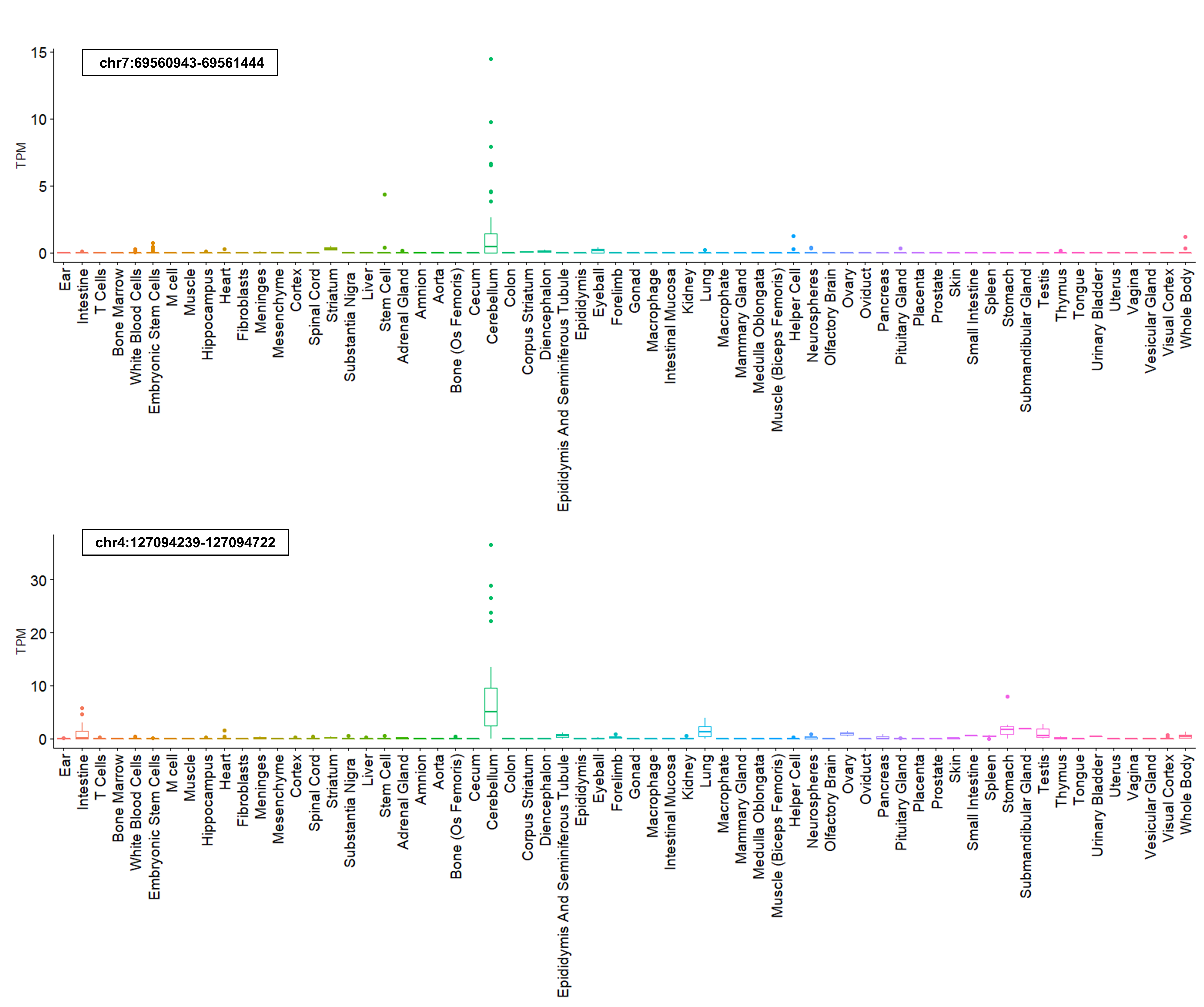

Supplement: Supplementary file 3 — Supplementary Material 3 [file 12864_2023_9368_MOESM3_ESM.png]

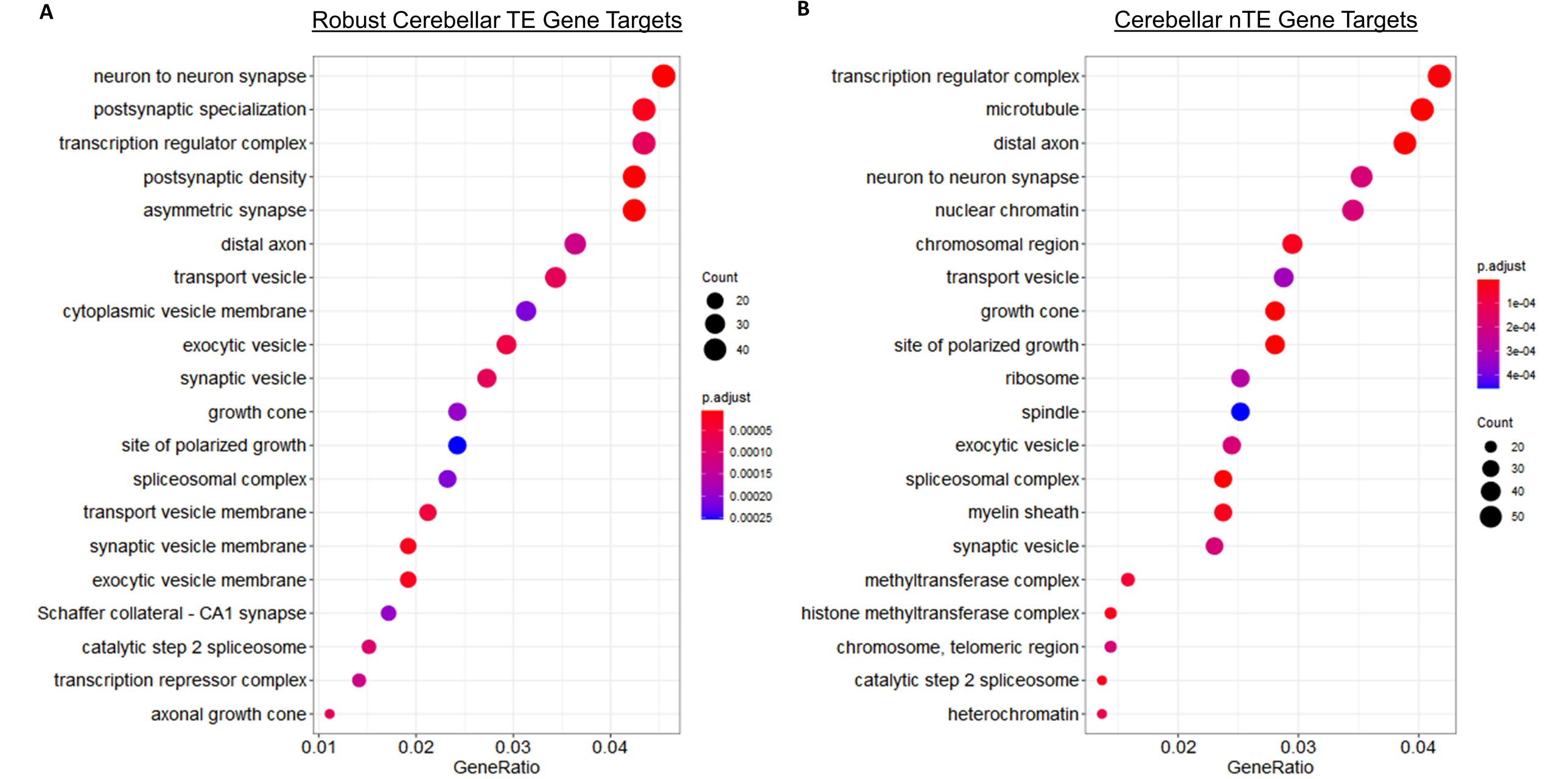

Supplement: Supplementary file 4 — Supplementary Material 4 [file 12864_2023_9368_MOESM4_ESM.png]

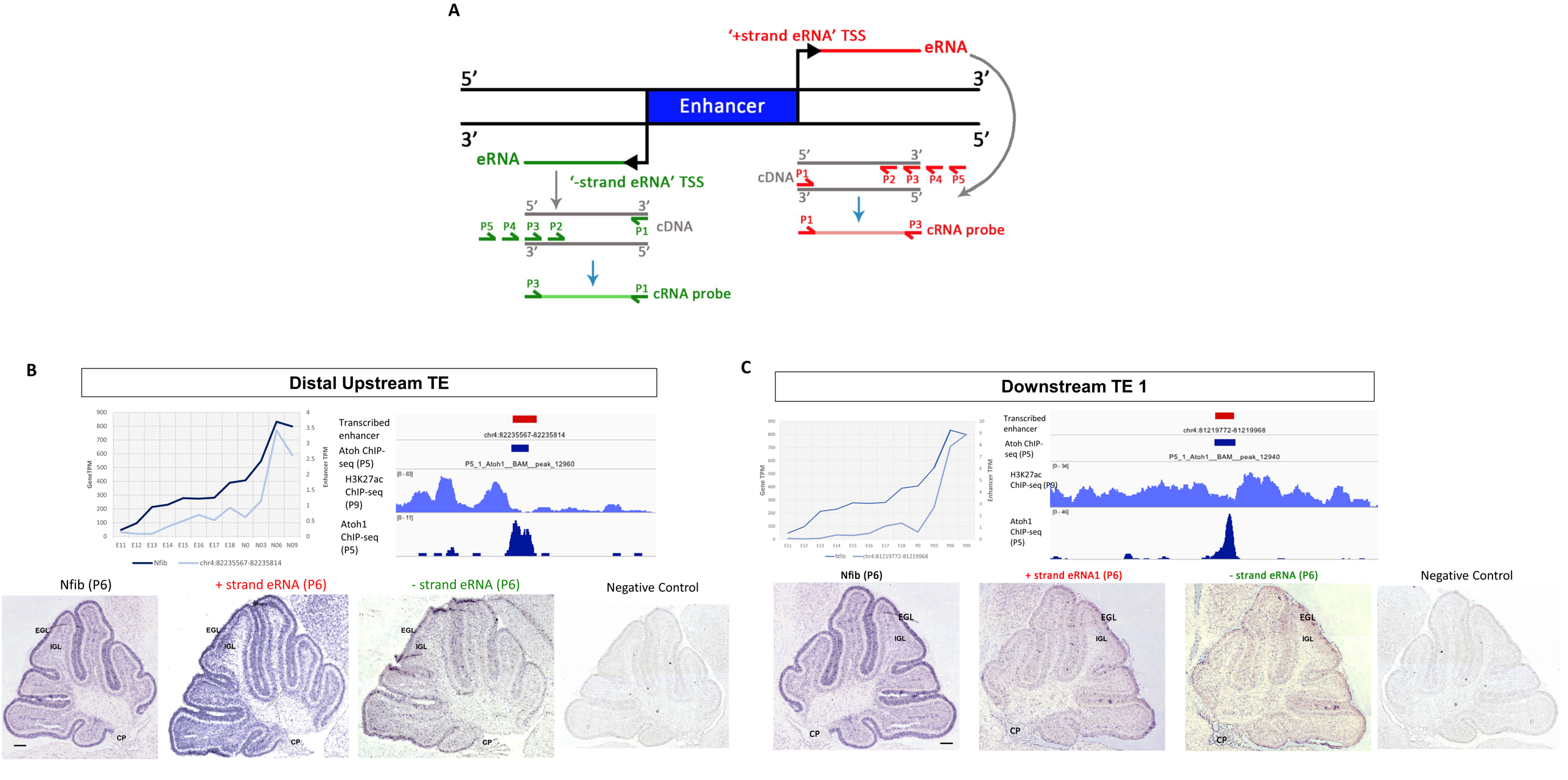

Supplement: Supplementary file 20 — Supplementary Material 20 [file 12864_2023_9368_MOESM20_ESM.png]
