# Supplementary material for: Identification and characterization of transcribed enhancers during cerebellar development through enhancer RNA analysis
Source: BMC Genomics. 2023 Jun 26;24:351. doi: 10.1186/s12864-023-09368-4 (PMC10291752; doi:10.1186/s12864-023-09368-4)
Supplement: Supplementary file 19 — Supplementary Material 19 [file 12864_2023_9368_MOESM19_ESM.pdf]

Supplementary Figure Legends

Supplementary Figure 1. Comparison of eRNA expression and histone modifications between robust and non-robust cerebellar enhancers. A) Line plot displaying the average expression of robust and non-robust cerebellar TEs at all examined time points during cerebellar development. The x-axis displays the normalized expression level in transcripts per million (TPM), while the y-axis represents stages of murine development. T-tests were conducted to examine the difference between mean expression between robust and non-robust TEs at each time point. **: <= 0.01, ***: <= 0.001, ****: <= 0.0001. **B)** Profiles of mean normalized CAGE-seq counts for robust cerebellar TEs (left column) and non-robust cerebellar TEs (right column) in the developing cerebellum at E12, P0 and P9. **C)** Profiles of normalized H3K4me1 ChIP-seq signal (top) and H3K27ac ChIP-seq signal (bottom) for robust cerebellar TEs relative to the centre of these elements. Average signal for robust cerebellar TEs (blue) and non-robust cerebellar TEs (green). **D)** Table displaying overlap of all cerebellar TEs and robust cerebellar TEs with publicly available ChIP-seq and DNase-seq datasets from P7 mouse cerebella.

Supplementary Figure 2. Expanded analysis of enhancer activity and eRNA expression after k-means clustering. A) Line plots displaying average normalized eRNA expression (green) and H3K27ac signal (pink) at E12, P0 and P9 for robust cerebellar TEs in k-means clusters 1 (left), cluster 2 (middle) and cluster 3 (right). **B)** Cluster plot of k-means analysis of non-robust cerebellar TEs. Four clusters are defined and the percentage of variance for dimensions 1 and 2 are 14.9% and 12.3% respectively. **C)** Line plots showing the average z-score normalized expression values (average normalized expression) over time for Cluster 1 (top), 2 (middle), 3 (middle), and 4 (bottom).

Supplementary Figure 3. eRNA transcription across mouse tissues. Boxplots showing eRNA expression for all FANTOM5 mouse tissues at tissue specific robust cerebellar TEs chr7:69560943-69561444 (top) and chr4:127094239-127094722 (bottom).

Supplementary Figure 4. Gene ontology analysis of putative gene targets of robust cerebellar TEs. A-B) Gene ontology enrichment analysis for cellular components of robust cerebellar TE targets (**A**) and non-transcribed targets (**B**). Gene ratio, which is represented on the x-axis, is the ratio between the number of robust cerebellar target genes within a given GO term and the total number of target genes. The number of genes within that GO term (Count) is signified by the dot size and the adjusted p-value (p.adjust) is represented by dot color.

Supplementary Figure 5. Putative gene target analysis for non-robust cerebellar TEs. A) Histogram showing the Pearson Correlation Coefficient of the most correlated gene target for all non-robust cerebellar TEs. Red line shows the cut off for significantly correlated gene target (p-value < 0.05). P-values were determined using a two-tailed t-test. **B)** Bar plot displaying the results of Gene Ontology enrichment analysis for the putative gene targets of non-robust cerebellar TEs. The x-axis displays Gene Ratio, which is the number of genes in the GO category divided by the total number of queried genes. The color scale displays the adjusted p-value (p.adjust).

Supplementary Figure 6. Analysis of robust cerebellar TEs with the same putative target gene. A) Donut plot displaying the distribution of robust cerebellar TEs with the same target gene in either 1 cluster, 2 clusters or 3 clusters. **B)** Histogram showing the average distance between robust cerebellar TEs from different k-means clusters with the same putative target gene The y-axis indicates the number of TEs with the same putative target gene and the x-axis represents the distance between the TEs predicted to regulate those target genes.

Supplementary Figure 7. *In situ* hybridization of eRNAs transcribed from robust cerebellar TEs predicted to regulate Nfib. A) PCR tiling strategy for ISH probe generation for eRNA transcripts. To identify PCR primers to amplify ISH probes, primers were designed every 100bp downstream of eRNA TSS. The primers from the longest successful PCR amplicon were used for probe amplification. **B-C)** Spatial eRNA expression analysis for Nfib, Distal Upstream TE (B), and Downstream TE 1 (C). Upper left panel: Expression pattern (TPM) for Nfib and TE throughout cerebellar development. Upper right panel: IGV Browser tracks showing transcribed enhancer (TE) location, Upstream TE H3K27ac ChIP-seq signal at P9 and Atoh1 ChIP-seq peak location and signal at P5. Lower panel: ISH at P6 for Nfib, Upstream TE "-" strand eRNA and "+" strand eRNA. EGL: External granule layer, IGL: Inner granule layer, CP: choroid plexus.
